# Enhancement of soil microbial community stability by earthworms and collembolans in soil from abandoned coal mine lands

**DOI:** 10.3389/fmicb.2026.1636784

**Published:** 2026-02-03

**Authors:** Junli Jia, Linghui Chen, Qian Liu, Kai Wang, Kang Zhao, Xiaoyu Ren, Xue Gao, Jianmei An

**Affiliations:** School of Life Science, Shanxi Province Engineering Research Center of Protection and Utilization of Endemic Animal Resources, Shanxi Normal University, Taiyuan, Shanxi, China

**Keywords:** edaphic macro-microfauna, microbial community co-occurrence network, mine soil rehabilitation, soil fauna, soil microbiome

## Abstract

Soil fauna play a critical role in the restoration of abandoned mining areas, uniquely contributing to soil formation, development, and the rehabilitation of degraded soils. This role is intricately linked with miwcrobial activity. Previous research has mainly concentrated on the direct effects of soil fauna on the physicochemical properties of soils in abandoned sites, often overlooking their indirect impacts on soil ecological functions via changes in soil microbial communities. This study undertakes a microcosm experiment by introducing soil fauna into the soil from coal mining abandoned lands to explore shifts in microbial communities. Results demonstrate that collembolan treatments significantly reduce fungi abundance, whereas earthworm treatments notably decrease the Shannon and Chao1 index for both bacterial and fungi communities. Soil fauna treatments modify the structure and composition of microbial communities, with more distinct differences in fungi community structures. Additionally, various soil fauna treatments markedly change microbial interactions; earthworm treatments impact microbial communities more than collembolan treatments, and combined treatments (EC) are more effective in enhancing microbial community stability compared to individual treatments (C, E). Network analysis has identified key microbial taxa that are positively correlated with soil fauna abundance, suggesting that future management strategies could manipulate key microbial taxa through soil fauna to enhance the restoration of soil ecological functions. These findings offer a detailed understanding of the dynamics of microbial communities under biotic interactions, essential for the ecological restoration of soils in abandoned mining areas.

## 1 Introduction

Coal mining rapidly impairs the integrity of terrestrial ecosystems, leading to extensive wastage of land resources. Concurrently, the soil structure, its capacity to retain water and nutrients, and the associated biological communities are almost completely degraded (Xin et al., 2023). Globally these wastelands already exceed 6.7 × 10^6^ hectares; China alone contains 2.88 × 10^6^ hectares (Wang, 2020; Young, 1988; Shu et al., 2000). Coal gangue is a primary industrial solid waste generated concomitantly during coal mining and processing. It is primarily composed of claystone, sandstone, and other lithologies, while also containing substantial concentrations of heavy metal ions. Given its large discharge volume, coal gangue not only poses risks of land occupation but also facilitates the leaching of heavy metals into the soil. This leaching event triggers a sharp decline in soil organic matter, alongside an increase in soil pH and total phosphorus content. Collectively, these edaphic alterations induce persistent toxicity, which inhibits the growth of plants, soil fauna, and soil microorganisms (He et al., 2024; Zhang D. E. et al., 2025; Zhang L. J. et al., 2025; Mao et al., 2020; Wang et al., 2019; Wang et al., 2013; Wang, 2011). Consequently, abandoned mine lands represent extreme ecological risk: simultaneously contaminated, eroded and biologically sterile, they pose a major challenge to any restoration strategy (Feng et al., 2021; Zhang D. E. et al., 2025; Zhang L. J. et al., 2025).

Because coal-mine disturbances rapidly disrupt soil physicochemical properties, the abundance and activity of key soil fauna such as earthworms and springtails—whose ecological functions depend on a stable soil matrix—are among the most sensitive indicators of ensuing ecological risk. As pioneering “biological engineers” on barren mine spoils, earthworms and collembolans quickly establish and drive soil recovery (Zhu et al., 2011; Mo et al., 2014; Ye et al., 2025). Earthworms (Oligochaeta) ingest and redox-transform heavy metals (Liu et al., 2009), excrete micro- and nano-plastics along macropore networks (Mo et al., 2023), and create enduring burrows that aerate and drain compacted substrates (Xu, 2019); collectively these activities raise porosity, organic-matter turnover, nutrient retention, and microbial activity (Hu et al., 2004; Sharma et al., 2017; Zheng and Li, 2019). Research has demonstrated that introducing earthworms to abandoned lands not only improves the soil’s physicochemical properties, increases soil porosity, and preserves nutrients, but also alleviates soil heavy metal pollution, thus fostering ecological recovery (Ge et al., 2001; Gao and Luo, 2005; Zheng and Li, 2019; Boyer and Wratten, 2010). Collembolans (Collembola), the most abundant detritivore arthropods, fragment litter, accelerate decomposition, and—especially when combined with organic amendments—rapidly increase soil organic carbon and nutrient cycling in reclaimed tailings (Li, 2014; Li and Zhu, 2015; Saifutdinov et al., 2020). Together, these two ubiquitous taxa ameliorate structure, fertility, and below-ground equilibrium, providing an essential ecological bridge for revegetation of derelict mine soils.

The capacity of soil fauna to drive the aforementioned functions hinges on the mutualistic, mutually reinforcing cascade effects that emerge from their intimate interactions with soil microorganisms. Both entities coexist in the soil and are intricately connected via the soil food web. Soil fauna affect the quantity, activity, composition, and function of soil microbial communities in various ways, such as enhancing the microhabitat (e.g., through defecation, burrowing, and soil stirring), increasing the surface area of organic matter, consuming it directly, and transporting and dispersing microbes (Li et al., 2013; Rossi et al., 2006; Wang G. H. et al., 2016; Wang J. et al., 2016). By re-engineering the physical, chemical and biological fabric of mine spoils, soil fauna act as catalysts of microbial reassembly and function. Earthworm burrows and collembolan hopping create stable biopores and micro-aggregates that simultaneously raise water-holding capacity and O₂ flux, favoring microbial recolonisation (Liu et al., 2025; Cao et al., 2015; Frouz et al., 2013). During passage through the gut, organic substrates are enriched 2–5-fold in C, N, P and S and are inoculated with a distinct microbiome; the resulting casts become nutrient-dense, metal-passivated microsites that accelerate local biogeochemical turnover (Kang and Wu, 2021; Wang et al., 2022; Hao et al., 2022). Faunal vectors also disperse metal-tolerant or plant-growth-promoting taxa while selective grazing suppresses pathogens and re-balances the bacterial:fungal ratio (Sun et al., 2021). These feedbacks heighten enzyme activity, generate nutrient hot-spots that facilitate pioneer plant establishment, and initiate a positive vegetation–fauna–microbe loop that propagates across the degraded landscape. In micro-plastic-contaminated tailings, for example, earthworm introduction restored arbuscular-mycorrhizal fungal diversity and tripled pepper biomass, demonstrating the capacity of fauna to re-activate indigenous mutualists and translate microbial functions into plant recovery (He, 2020; Wu et al., 2024). The presence of soil fauna directly boosts microbial activity and indirectly increases the availability of numerous nutrients, with multispecies systems proving more effective than single-species systems in improving soil properties and quality (Zhu et al., 2011). Previous studies have provided little insight into how soil fauna perturb microbial communities in coal-mine substrates, leaving a critical knowledge gap in the ecological restoration of post-mining landscapes.

Soil microbes, the “decomposers” within the soil ecosystem, play an irreplaceable role in enhancing soil fertility, transforming mineral elements, metabolizing organic matter, degrading pollutants, and contributing to soil structure, thus impacting the cycling and flow of materials and energy within soil ecosystems (Du et al., 2022; He et al., 2009; Hu and Li, 2018; Yi and Li, 2018). Mining activities alter the microenvironment of soil in abandoned lands, leading to changes in the quantity, diversity, and distribution of soil microbes. These alterations can be indicated by variations in microbial activity, community structure, and diversity, reflecting the soil’s quality and health (Insam et al., 1996; Knight et al., 1997; Jiang et al., 2000).

Although the influence of soil animals on the physical and chemical properties of soil has been extensively studied by scholars, the interaction of soil microorganisms is indispensable in this process, and the understanding of the role of soil microorganisms in this process is lacking. The influence of direct or indirect activity of soil animals on soil microbial community leads to the change of soil physical and chemical properties. Especially, there is limited understanding of the interaction between microorganisms in abandoned coal mines and the interaction between animal disturbance and microorganisms.

Here, we address this knowledge gap by experimentally introducing two ubiquitous soil-engineering taxa—earthworms and collembolans—into coal-mine wastelands. Our overarching aim is to quantify how faunal re-establishment restructures soil bacterial and fungal communities and rewires their biotic interactions, thereby providing a mechanistic pathway for accelerating below-ground recovery. We hypothesize that, (1) soil-faunal addition will significantly affect microbial community structure; (2) animal-mediated enhancement of microbe—microbe stability and connectivity will increase community resilience and catalyze ecosystem restoration in derelict post mining soils.

## 2 Materials and methods

### 2.1 Study area

This study was conducted at Baijiazhuang Coal Mine in Wanbailin District, Taiyuan City, Shanxi Province, China (37.81777 °N, 112.40768 °E). However, the coal resources in this study area had been depleted and mining had stopped in 2016. Nevertheless, a large amount of coal gangue and waste slag generated during mining production still exist, causing great damage to the land, and no ecological restoration measures have been taken for this abandoned mine. This study specifically targeted the disruptive effects of soil-faunal inoculation on soil microbial communities; detailed quantification of edaphic variables was beyond the present scope, and all physicochemical data were compiled from recent investigations conducted in the same study region. The city belongs to the northern temperate continental climate, with an average annual rainfall of 456 millimeters and an average annual temperature of 9.5 °C. The coal mine is located about 9 km west of the center of Taiyuan city, with an area of approximately 0.26 km^2^ and an altitude of 800–1,600 meter. The dominant soil types are mountain brown earth and brown earth-like soil, with overall poor soil quality. According to previous research findings: the contents of heavy metals (e.g., Pb, Cd, and Cr) in the soil exceed the heavy metal background values of Shanxi Province; the soil has a higher sand content; the soil pH ranges from 8.07 to 8.54, which is higher than that of the surrounding areas unaffected by coal mining activities; additionally, the soil organic matter (SOM) content in this region ranges from 9.25 to 57.48 g kg^−1^, which is significantly lower than that of the surrounding areas; the soil organic carbon (SOC) content is also lower than that in other regions (Duo, 2024; Li, 2023; Xie et al., 2022).

### 2.2 Sample collection

**Figure 1:**
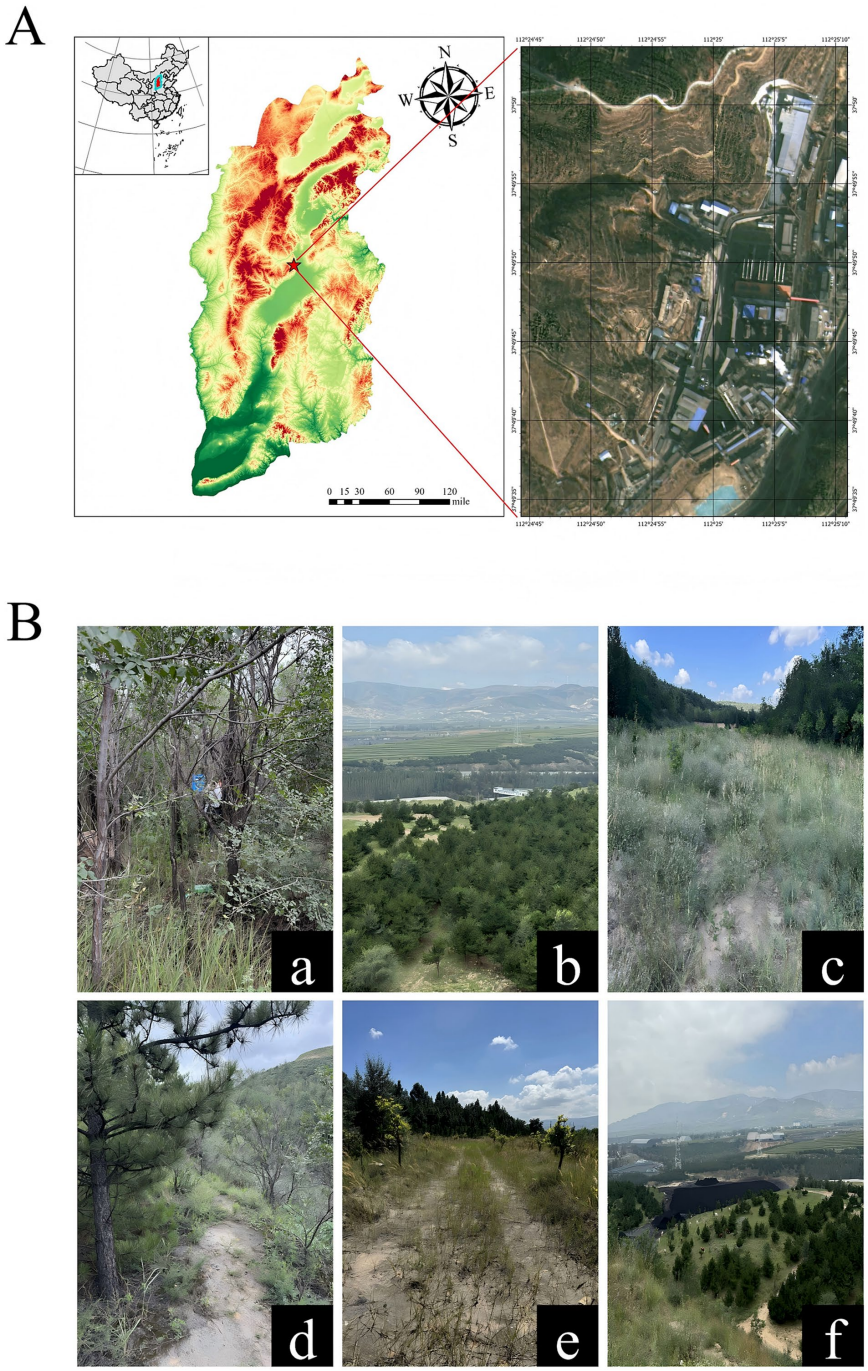
Geographical location of the sampling area and representative habitat characteristics of the study site. **(A)** Displays the exact geographical location of the Baijiazhuang Coal Mine within the study region, where the five-pointed stars specifically denote the precise positions of all sampling sites established for soil in this study. **(B)** Illustrates the representative habitat conditions of the sampling area, including key features such as dominant vegetation types, soil substrate characteristics, and typical landscape elements associated with coal mining activities. **(a)** Represents the typical habitat of the sampling site. **(b)** Shows the operating coal mining equipment visible in the distance. **(c)** Depicts the typical shrub vegetation at the sampling site. **(d,e)** Illustrate the soil substrate conditions during the post-coal mining restoration stage. **(f)** Displays the accumulation state of coal seams.

In May 2023, we conducted sampling at the Baijiazhuang coal mine (Figure 1). We select the same place with the same habitat, adopt the five point sampling method, set up 10 cm × 10 cm sampling points, collect waste surface layer (0–10 cm) soil with a shovel, a total of 32 soil samples, put them into bags, mix them evenly, and transport them back to the laboratory (Jacques et al., 2021; Lin et al., 2012). Fresh soil samples are mixed, air dried, and thoroughly homogenized through a 2 mm sieve to remove impurities such as stones and grass roots, and then stored at −20 °C. Immediately conduct indoor microcosm experiments to maintain the activity of soil microbial communities.

### 2.3 Establish a laboratory microcosm

**Figure 2:**
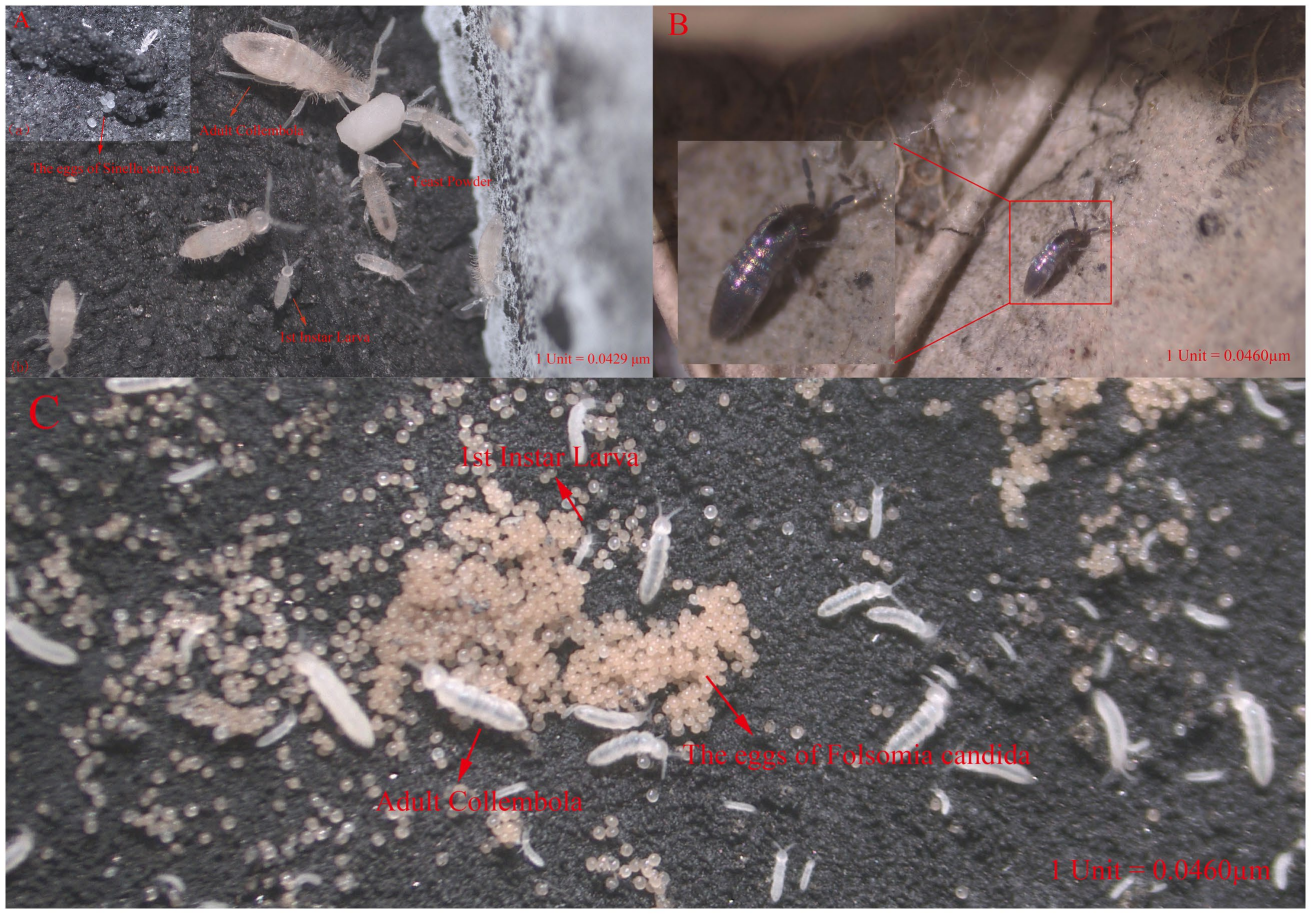
Culture status of three Collembola species under laboratory conditions. **(A)** Culture status of *Sinella curviseta*. The black substrate in the background is a custom-made rearing medium for Collembola prepared in the laboratory. **(a)** Eggs and newly hatched 1st instar larvae of *Sinella curviseta*. **(b)** Larvae, juveniles, and adults feeding on yeast granules. **(B)** Survival status of *Lepidocyrtus cyaneus* adults on humus. **(C)** Eggs, larvae, and adults of *Folsomia candida*.

Earthworms (*Eisenia fetida*) were purchased from Wangjun Earthworm Farm in Jurong City, Jiangsu Province, China, with an average weight of 0.35 ± 0.2 g. Before the experiment, they were acclimatized for 7 days in the collected abandoned land soil. The collembolans *Sinella curviseta* and *Lepidocyrtus cyaneus*, common local species from Shanxi Province, China, were captured in the field and subsequently cultured and expanded in the laboratory for use in the experiments. Additionally, the ecotoxicological model species *Folsomia candida* was purchased from the Huitong Forest Experiment Station in Hunan Province, China, and bred in our laboratory for some time before use in the experiments. Collembolans were synchronously cultured under controlled conditions. Fifty adults of uniform size were placed in a Petri dish containing a culture medium of calcium sulfate, water, and activated charcoal (8:6:1). After egg deposition, eggs were transferred to fresh medium using a fine brush. Larvae were maintained under the same conditions, with twice-weekly supplementation of distilled water and yeast (Angel Yeast Co., Ltd.), while temperature and humidity were regulated throughout the incubation period (Goto, 1960; Yang, 2008). The synchronized culture conditions of the three Collembola species are illustrated in Figure 2.

Microcosm experiments were conducted in 1000 mL transparent beakers with a diameter of 10.5 cm and a height of 14.5 cm. The mouth of each beaker was sealed with a plastic film perforated with small holes, and secured with rubber bands to prevent the escape of soil fauna while ensuring adequate gas exchange with the external environment.

### 2.4 Experimental design

The experiment included four treatments: separate soil animal treatment with only earthworms (E), separate treatment with only collembolans (C), combined treatment with earthworms and collembolans (EC), and control treatment (CK). Each treatment was repeated eight times, for a total of 32 samples. For each beaker, add 800 grams of soil and maintain the soil moisture at approximately 80% using distilled water (measured with a soil moisture meter). Earthworms are washed in tap water to minimize contamination from other soil animals, then gently wiped dry on a cloth, weighed, and placed on the soil surface. In earthworm treatment (E), 10 grams of fresh weight earthworms were added, which is equivalent to adding 0.0125 grams of earthworms per gram of soil (calculation method: 10 grams of earthworms/800 grams of soil), equivalent to 25–27 individuals of mixed size and age (Xiang et al., 2023). For the treatment of collembolans (C), a mixture of three types of collembolans with 100 individuals each taken from laboratory culture was added to the soil surface, equivalent to adding 0.125 collembolans per gram of soil (calculation method: 100 collembolans/800 grams of soil) (Liu et al., 2008). The combined treatment (EC) simultaneously added the above-mentioned quantities of earthworms and collembolans (Jacques et al., 2021). The microcosm device is placed in a constant temperature (20 ± 1)°C incubator and kept in the dark for two months, with regular water addition to maintain soil moisture at around 80% without any supplementary food amendment.

### 2.5 Extraction of soil fauna

After the experiment, to assess the survival of soil fauna, earthworms were collected by hand sorting, washed, and excess water was removed before they were weighed and counted. Collembolans were extracted using the Tullgren funnel method^1^ by placing the soil from the collembolan treatments (C and EC) under a light for three days. The Tullgren funnel, a standard method for extracting soil fauna (Hopkin, 2007), uses a 40 W bulb suspended above the soil in the funnel to create a temperature gradient that encourages soil fauna to migrate downwards until they fall into a collection fluid (70% alcohol). Collembolans preserved in alcohol were accurately identified to species, and no other soil fauna groups were detected during the identification process.

### 2.6 Microbial DNA extraction and fluorescent quantification of 16SrRNA and ITS genes

We amplified operational taxonomic units (OTUs) from total soil DNA (16S/ITS), which represent the complete soil microbiome (Xu et al., 2024; Zhou et al., 2024). Genomic DNA was extracted from 0.5 g of soil samples using a DNA kit provided by Biomarker Technologies Co., Ltd. The quality of the extracted DNA was checked using 1% agarose gel electrophoresis, and the concentration and purity of the DNA were assessed using a spectrophotometer (BioTek, United States). The abundance of bacterial 16S rRNA and fungi ITS genes in the samples was detected using real-time fluorescent quantitative PCR with a QuantStudio 3 Real-Time PCR System (Thermo, United States) (Huang et al., 2016; Clemmensen et al., 2016; Liu et al., 2021; Zhang et al., 2022; Wang et al., 2023; Li et al., 2025). Primers used were 1369F/1541R for bacteria and gITS7/ITS4 for fungi. The PCR reaction volume was 15 μL, containing 1 μL DNA (~10 ng), 0.4 μL of 10 μM primers, and 7.5 μL SYBR Green Premix (Takara, Japan). PCR cycling conditions were 95 °C for 5 min, 56 °C for 30 s, and 72 °C for 30 s. Each sample was measured in triplicate, and all qPCRs included no-template negative controls, with fluorescence data collected at the end of the annealing phase. Standard curves were generated by diluting plasmids containing the 16S rRNA and ITS genes (ranging from 10 to 10^8^) to calculate the gene copy numbers in the samples.

### 2.7 High-throughput sequencing and data analysis of 16SrRNA and ITS genes

High-throughput sequencing and sequence processing of 16S rRNA and ITS genes were carried out by the sequencing platform of Biomarker Technologies Co., Ltd., using the same primers as in the fluorescent quantitative PCR. The basic procedure for sequencing and analysis was as follows: amplicons were sequenced on the Illumina NovaSeq 6000 platform in paired-end mode (2 × 250 base pairs). Low-quality sequences were filtered out using Trimmomatic v0.33 (sliding window: 50:20). Paired-end sequences were merged using Usearch v10. Chimera sequences were removed using UCHIME software. High-quality 16S rRNA sequences were denoised using the DADA2 method in QIIME2 2020.6 software to obtain ASVs. High-quality ITS sequences were processed using Usearch v10 to differentiate operational taxonomic units (OTUs) at a 97% similarity threshold. Representative sequences for each ASV and OTU were aligned against the UNITE database for phylogenetic analysis of bacterial and fungi 16S rRNA and ITS sequences. To compensate for sequencing depth differences, the number of 16S rRNA gene sequences per sample was normalized to 37,599 and ITS gene sequences to 62,241.

### 2.8 Statistical data analysis

Pairwise comparisons of earthworm biomass and collembolan numbers were conducted using t-tests with SPSS (version 26.0; SPSS, Chicago, IL, United States) (Wu et al., 2019). Microbiome data analysis was performed using R (version 4.2.2) (Liu et al., 2024). The abundance of bacteria and fungi was log_10_-transformed and analyzed using one-way ANOVA with LSD tests (Herrmann and Jürgen, 2018). The Shannon and Chao 1 index of bacterial and fungi communities at the OTU level were calculated and analyzed using pairwise *t*-tests to assess differences in mean abundance, Shannon index, and Chao 1 index under different treatments (Cho et al., 2021). Principal coordinates analysis (PCoA) was used to assess differences in the community structure of bacterial and fungi abundances, and non-parametric multivariate analysis of variance (PERMANOVA) was used to evaluate differences in community composition (Qin et al., 2022). Stacked bar charts were used to display community composition at different taxonomic levels, and Venn diagrams were used to analyze generalist and specialist species of bacteria and fungi under different treatments.

Ecological interactions between species in microbial communities and between microorganisms and soil animals are described by constructing molecular association networks using the Inter-Domain Ecological Network pipeline (IDENs) on the iNAP platform (Galaxy|iNAP^2^) (Feng et al., 2022). Initially, we retained only those OTUs/ASVs that were present in no fewer than seven replicates across the different soil-fauna treatments for inter-domain network construction. A filtered compositional table was then subjected to SparCC (Sparse Compositional Correlation) to estimate all pairwise associations. Intra-bacterial networks were generated by retaining associations whose Spearman correlation coefficient was at least 0.80 in absolute value and whose two-tailed *p*-value, after 1,000 permutations, fell below 0.05. Intra-fungal networks were constructed under the same significance threshold but with a minimum absolute correlation of 0.85 (Xing et al., 2025; Zhou and Wu, 2021; Shu et al., 2021). For the inter-domain network, we first retained only those OTUs/ASVs detected in no fewer than seven replicates across all soil-fauna treatments, then pruned the adjacency matrix to correlations whose absolute value reached 0.85 and whose two-tailed *p*-value remained below 0.05, yielding the final co-occurrence network. After network construction, global network attributes, network randomization, and outputs for visualization were performed on the platform, describing network topological features. Nodes were categorized into four types based on their within-module connectivity (Zi) and among-module connectivity (Pi): Peripherals (Zi ≤2.5, Pi ≤0.62), Module hubs (Zi >2.5, Pi ≤0.62), Connectors (Zi ≤2.5, Pi >0.62), and Network hubs (Zi >2.5, Pi >0.62), with Network hubs, Module hubs, and Connectors considered key nodes in the microbial network (Deng et al., 2012). Additionally, network robustness was characterized using a robustness metric, calculating robustness when 50% of random nodes were removed. Networks and modules were visualized in Gephi 0.10.1.

## 3 Results

### 3.1 Response of soil fauna to incubation

**Figure 3:**
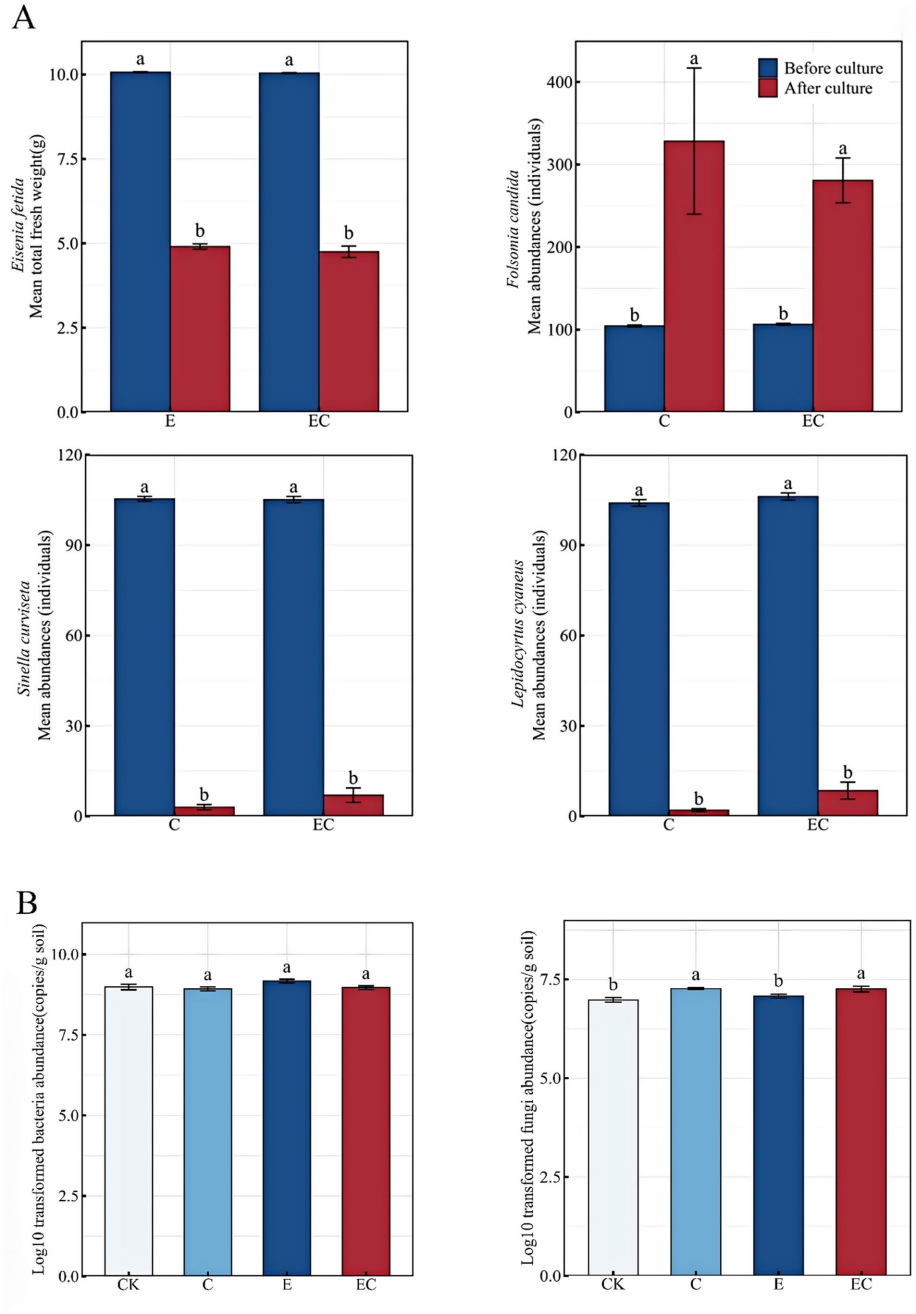
Abundance characteristics of soil animals and microorganisms. **(A)** Soil animal abundance characteristics under different treatments and after and before treatment. **(B)** Bacteria and fungi abundance characteristics under different treatments. Different letters represent significant differences (*p* < 0.05). CK: control, C: collembola present, E: earthworms present, EC: earthworms and collembola present. Error bars = SE.

After the 60-day incubation, the abundances of most soil fauna declined markedly, with decreases observed in the biomass of *Eisenia fetida* and the abundances of *Sinella curviseta* and *Lepidocyrtus cyaneus*. In contrast, the population of *Folsomia candida* increased significantly (Figure 3A).

### 3.2 Microbial abundance and community composition

#### 3.2.1 Abundance of bacteria and fungi

Fungal abundance differed significantly among treatments (*p* < 0.05), following the sequence EC > E > CK > C. In contrast, no significant difference in bacterial abundance was detected across treatments (*p* > 0.05) (Figure 3B and Supplementary Tables S1, S2).

#### 3.2.2 Community composition at phylum and genus levels

**Figure 4:**
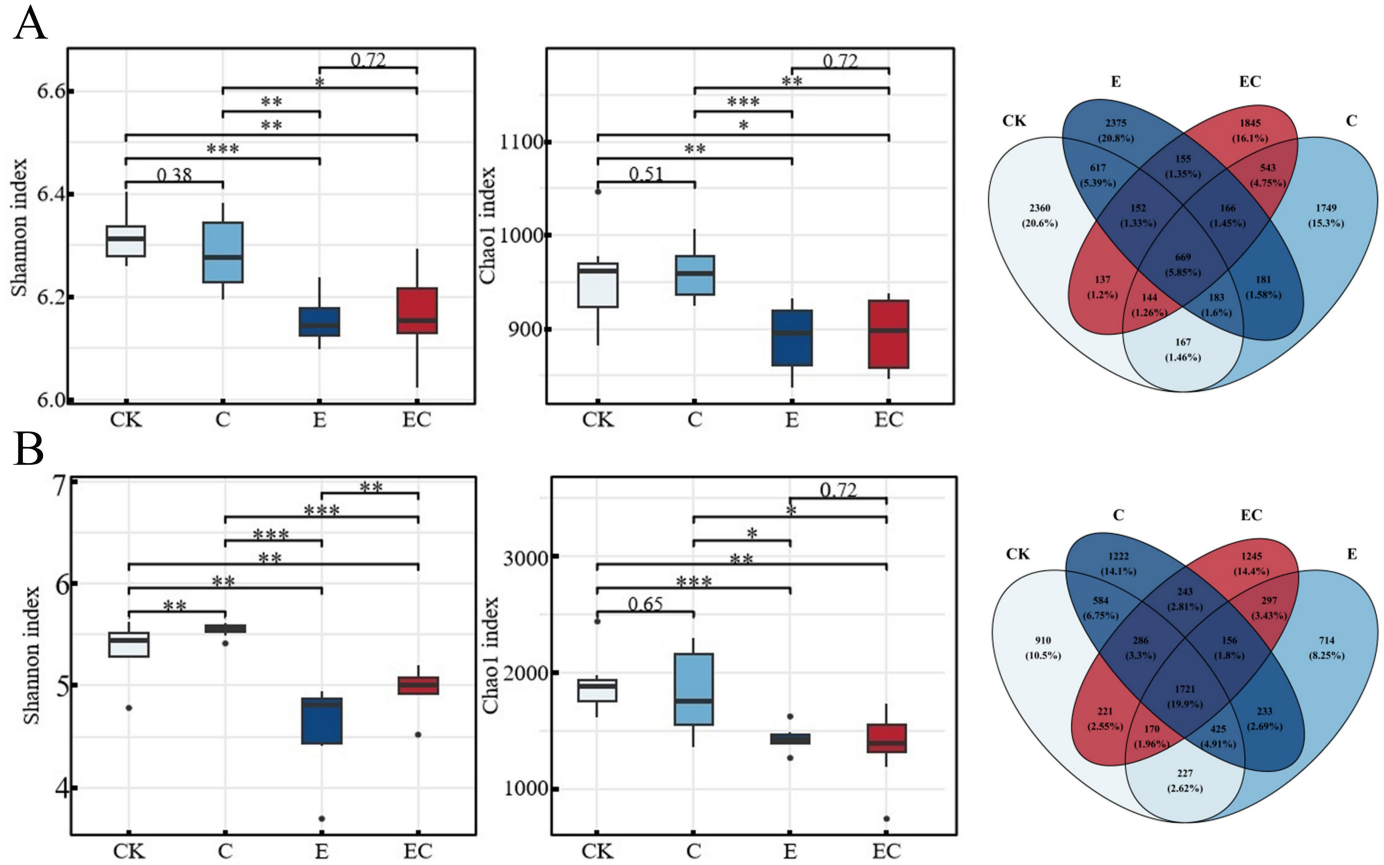
Microbial α diversity index and species distribution pattern characteristics. **(A)** Bacteria α diversity index and distribution pattern of bacteria species (ASV) under different treatments. **(B)** Fungi α diversity index and distribution pattern of fungi species (OTU) under different treatments. * represent *p* < 0.05, ** represent *p* < 0.01, *** represent *p* < 0.001.

A total of 4,498 bacterial ASVs and 4,042 fungal OTUs were identified. Treatments CK and C shared a high proportion of unique bacterial ASVs (20% of the total), while treatments C and EC shared 14% of unique fungal OTUs (Figures 4C,D).

The bacterial communities were predominantly composed of Actinobacteriota, Proteobacteria, and Acidobacteriota (Supplementary Figure S1A). Treatments E and EC were characterized by a higher relative abundance of Proteobacteria, whereas CK and C exhibited a greater prevalence of Acidobacteriota, Gemmatimonadetes, and Myxococcota (Supplementary Figure S1C). Fungal communities were dominated by Ascomycota, Mortierellomycota, and Basidiomycota (Supplementary Figure S1B). Treatment C showed the highest abundance of Ascomycota, while Mortierellomycota was most prevalent in treatment E (Supplementary Figure S1D).

**Figure 5:**
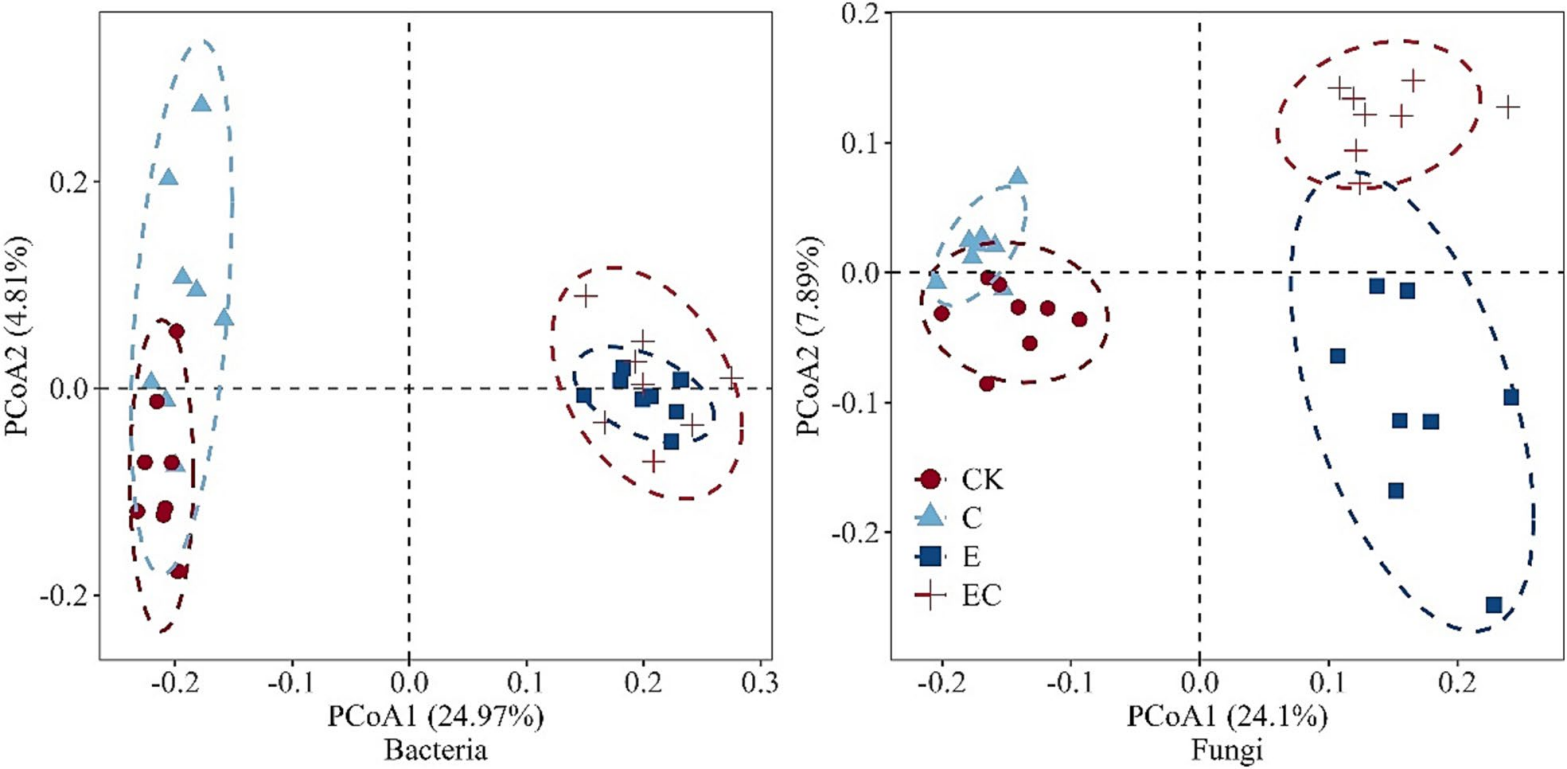
Principal coordinates analysis (PCoA) of bacteria and fungi community structure at different treatments. Different colors represent different treatments.

Principal coordinates analysis (PCoA) based on Bray–Curtis dissimilarity revealed a clear separation of microbial communities. Bacterial and fungal communities in CK and C clustered together, forming a distinct group from those in E and EC. PERMANOVA confirmed that these compositional differences were statistically significant (*p* < 0.05) (Figure 5; Supplementary Figure S2 and Supplementary Tables S3, S4). Notably, the separation among treatments was more pronounced for fungal communities than for bacterial communities.

Mantel tests further indicated significant positive correlations between the presence of soil fauna and key microbial genera (Supplementary Table S7). Earthworms were positively correlated with bacterial genera Var*iovorax*, *Arenimonas*, and *Blastococcus*, and with fungal genera *Condenascus*, *Acrophialophora*, and *Pyrenochaeta*. Collembolans were positively correlated with bacterial genera *Labrys*, *Dongia*, *Endozoicomonas*, and *Agromyces*, as well as fungal genera *Acrophialophora*, *Basidioascus*, and *Glaciozyma*.

### 3.3 Microbial community diversity

Both bacterial and fungal communities showed significant variations in α-diversity indices among treatments (*p* < 0.05). Treatments CK and C exhibited substantially higher Shannon diversity and Chao1 richness indices for both bacteria and fungi compared to treatments E and EC (Figures 4A,B).

### 3.4 Architecture and stability of microbial co-occurrence networks

**Figure 6:**
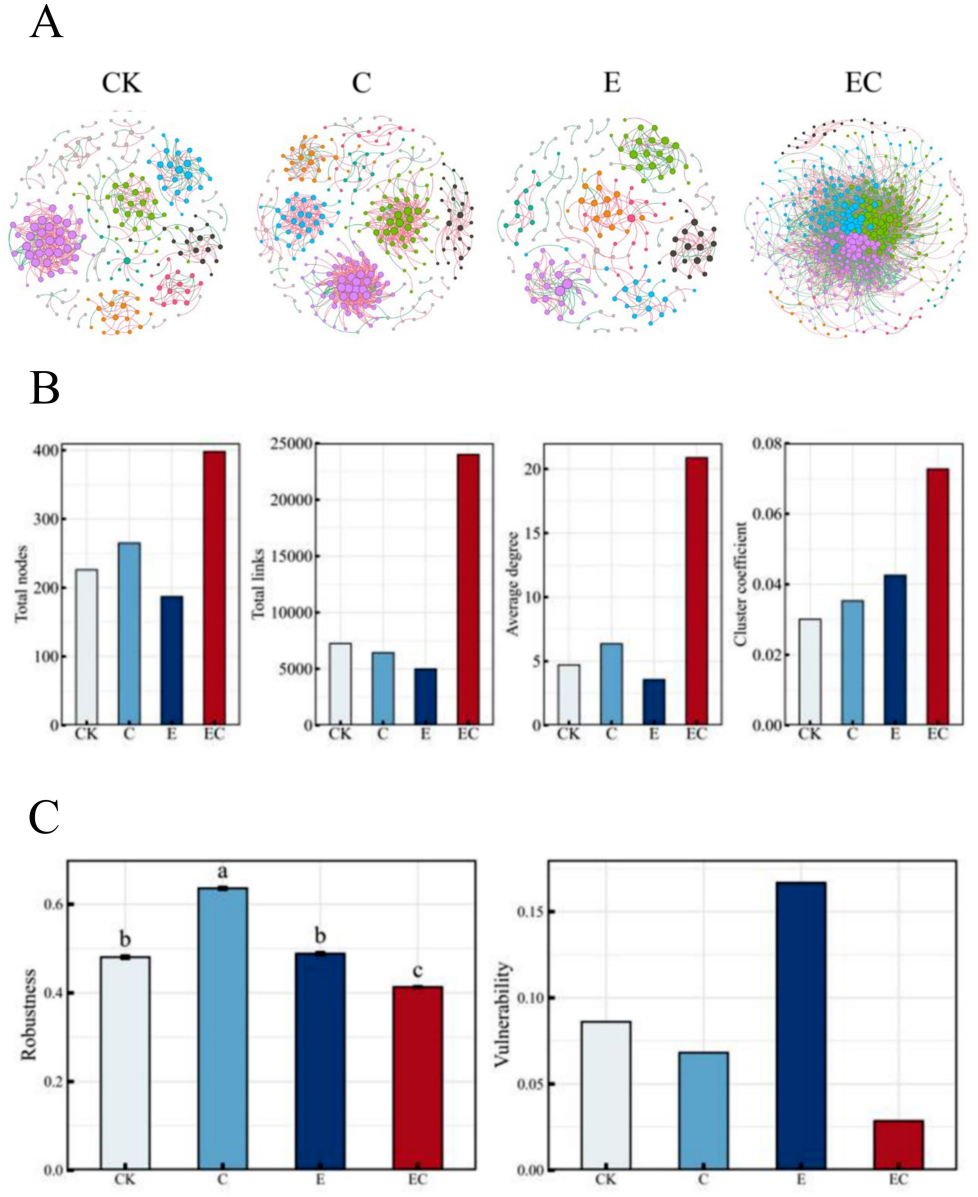
Microbial co-occurrence network pattern under different treatments. **(A)** Bacteria-fungi co-occurrence network characteristics under different treatments. The top eight modules in abundance are represented by different colors and other modules are represented by gray, the size of the nodes represents degrees, and the color of the edges represents positive or negative correlations, with red representing positive correlations and green representing negative correlations. **(B)** Topological characteristics of the microbial symbiotic network under different treatments. **(C)** Stability characteristics of the microbial symbiotic network under different treatments.

The bacterial-fungal association networks demonstrated that different treatments significantly altered their structural architecture (Figure 6A). The combined fauna treatment (EC) yielded the most complex network, possessing the largest number of nodes and edges, indicative of a hyperconnected community (Figure 6B).

Quantitative analysis revealed that the EC treatment substantially enhanced network stability, increasing robustness by 32% and reducing vulnerability by 19% compared to controls (Figure 6C). Topological analysis confirmed that the empirical networks exhibited higher connectivity, nestedness, and modularity than their corresponding random networks (Supplementary Table S5). Under the same threshold, the EC network showed higher clustering coefficients and average degree. Furthermore, over half of the connections in the E and EC networks were negative, suggesting that competitive or antagonistic interactions dominated within these microbial communities.

Key node analysis identified a total of 66 core species across all treatments. The number of key nodes (module hubs and connectors) was highest in the EC treatment, which contained 53 connector hubs and six module hubs, compared to only one in CK, two in C, and four in E (Supplementary Table S6). Sub-networks based on soil animal-related nodes further demonstrated that treatments C and EC retained more nodes and a higher proportion of positive correlations (Supplementary Figure S3).

## 4 Discussion

### 4.1 Response of soil Fauna to the restoration environment

Consistent with the observations from our 60-day incubation experiment, the populations of most soil fauna taxa exhibited distinct asynchronous trajectories: the biomass of *Eisenia fetida* and abundances of *Sinella curviseta* and *Lepidocyrtus cyaneus* declined markedly, while the population of *Folsomia candida* increased significantly (Figure 3A). These divergent responses can be explained in the context of the study site’s soil characteristics: the soil matrix used in this research exhibits excessive heavy metal concentrations alongside low contents of soil organic matter (SOM) and soil organic carbon (SOC), which was reflected in the gradual resource depletion during the incubation period. Resource scarcity and heavy metal stress collectively constitute a “dual-filter” effect, jointly shaping the variations in soil faunal communities via trophic limitation and metabolic toxicity (Ge et al., 2001; Yan et al., 2019). Specifically, the decline of *Eisenia fetida* aligns with its known preference for fresher, nutrient-rich substrates (Hanc et al., 2020; Guo, 2024)—a resource that was limited in our microcosm system, constraining its survival and biomass accumulation. In contrast, *Folsomia candida* maintained a competitive edge due to its dietary generalism (enabling utilization of limited available resources) and high humidity tolerance (adapted to the microcosm’s conditions) (Ding, 2018), which directly explains its significant population growth. The reduced abundances of *Sinella curviseta* and *Lepidocyrtus cyaneus*, meanwhile, likely reflect their weaker tolerance to the combined stress of resource shortage and heavy metals, relative to the more adaptable *Folsomia candida*.

Notably, our microcosm lacked vertical refugia, a factor that amplified the stress imposed by resource scarcity and heavy metals (Briones, 2014, 2018). This ties to a practical implication for field restoration: supplementing surface litter (to provide both resources and refugia) could mitigate such top-down pressure on soil fauna, supporting more stable communities during restoration. Crucially, this taxon-specific response pattern also provides foundational context for interpreting our microbial community results (linked to Hypothesis 1): soil fauna-mediated effects on microbes are taxon-dependent, so the divergent faunal trajectories directly shape subsequent microbial community dynamics.

### 4.2 Fauna-induced changes in soil physicochemical properties

The introduction of soil fauna initiates a cascade of processes that ultimately modify the soil’s physical and chemical environment. Earthworms, through their burrowing and casting activities, are renowned for improving soil structure, aeration, and water infiltration (Yang et al., 2017). More critically, these physical modifications, combined with the digestive processing of organic matter, catalyze profound changes in nutrient cycling. Our findings suggest that the fauna-induced microbial shifts (detailed in subsequent sections) are a key mechanism driving these changes. As bacteria and fungi are the primary agents of decomposition and nutrient mineralization, any faunal-driven restructuring of the microbial community is expected to propagate to the soil physicochemical level, altering the activities of carbon, nitrogen, and phosphorus-transforming enzymes and accelerating organic matter turnover rates (Nelson et al., 2016; Kost et al., 2023). This functional link supports the potential of soil fauna as a viable bioremediation strategy for improving soil fertility in contaminated lands.

In our study, we focused primarily on the interactions between soil fauna and soil microbial communities, and thus did not conduct measurements of soil physicochemical properties. While our results reveal strong correlative patterns between these biotic components, the absence of soil physicochemical data limits our ability to quantify the causal chains linking specific faunal activities to changes in soil nutrient pools or physical structure. Accordingly, future studies should incorporate detailed determinations of key soil physicochemical properties (e.g., nutrient dynamics, texture, and cation exchange capacity)—this will enable the explicit elucidation of how faunal-microbial interactions mediate soil ecosystem functional changes through modifying substrate properties.

### 4.3 Effects of soil faunal addition on soil microbial community structure and genus-level assemblages

Our first hypothesis that soil-faunal addition would significantly affect microbial community structure is strongly supported by our results: soil fauna introduction drove distinct taxonomic restructuring of the soil microbiome, with clear separation of bacterial/fungal communities across treatments (PERMANOVA, *p* < 0.05; Figure 5; Supplementary Figure S2). Notably, this separation was more pronounced for fungal communities, highlighting fauna’s stronger regulatory effect on fungi.

While phylum-level analysis identified dominant taxa (e.g., Actinobacteriota/Proteobacteria in bacteria; Ascomycota/Mortierellomycota in fungi; Supplementary Figures S1A,B), genus-level assemblages uncovered treatment-specific patterns that directly reflect fauna-mediated effects. For bacterial communities, treatments E and EC exhibited elevated Proteobacteria abundance, aligning with Mantel test results that linked earthworms to bacterial genera Var*iovorax* (xenobiotic degradation), *Arenimonas* (nitrogen mineralization), and *Blastococcus* (recalcitrant organic matter decomposition; Supplementary Table S7) (Dong et al., 2022; Hou, 2021; Finkel et al., 2020), while CK and C treatments were dominated by *Acidobacteriota*/*Gemmatimonadetes* (soil organic matter transformation and the ability to efficiently decompose organic matter to obtain carbon sources), with collembolans correlating positively with genera *Labrys* (polysaccharide hydrolysis) and *Agromyces* (cellulose degradation; Supplementary Table S7) (Fawaz, 2013; Wang G. H. et al., 2016; Wang J. et al., 2016). In parallel, fungal genus patterns tracked with fauna treatments: Treatment C showed the highest Ascomycota abundance, matching collembolans’ gut-associated fungal taxa (Hao et al., 2022, 2024; Anslan et al., 2018) and collembolans’ positive correlation with *Acrophialophora* and *Basidioascus* (Supplementary Table S7), while Treatment E was dominated by *Mortierellomycota*, consistent with earthworm-mediated fungal dispersal (Vries et al., 2018; Xiang et al., 2019) and earthworms’ correlation with *Condenascus* (Supplementary Table S7). These genus-level shifts are likely driven by the “gut inoculation” effect: *Eisenia fetida*’s gut microbiome is enriched in Proteobacteria that explaining *Variovorax*/*Arenimonas* enrichment in E/EC treatments (Singh et al., 2015; Ma et al., 2017). And collembolans harbor Ascomycota-dominated gut fungi aligning with *Acrophialophora* elevation in C/EC treatments, and the 14% shared unique fungal OTUs between C and EC (Figure 4D) further support cross-fauna taxon dispersal in combined treatments (Anslan et al., 2018).

Critically, these genus-level changes are functionally meaningful: taxa like *Variovorax* (pollutant degradation) and *Labrys* (carbon cycling) are central to biogeochemical processes in our study’s heavy-metal contaminated soil, so fauna-induced shifts in microbial genera directly modulate soil functional capacity, linking to the practical value of faunal addition for contaminated soil restoration.

### 4.4 Alterations in soil microbial community diversity

Faunal activities also exerted a significant influence on microbial α-diversity, with effects varying by faunal group. Treatments including earthworms (E and EC) significantly lowered the Shannon and Chao1 indices for both bacteria and fungi compared to the control (CK). This decline in α-diversity can be attributed to the strong ecosystem engineering role of earthworms. Their movement and feeding create a more homogeneous soil environment and selectively enrich a subset of fast-growing, r-strategist microorganisms, thereby reducing overall community diversity (Yang et al., 2017; Sun et al., 2021). In contrast, the collembolan-only treatment (C) showed no significant difference in diversity indices from CK, suggesting that as smaller mesofauna with more limited dispersal and bioturbation capacities, their impact on overall microbial community diversity is less pronounced (Shao et al., 2015).

The direct feeding and indirect activities of soil fauna affected specific microbial groups, leading to changes in soil microbial community structures (Wang et al., 2022; George et al., 2017). Our findings imply that ingested microorganisms undergo a gut-mediated “selective sieve” within nutrient-rich faunal intestines, whereby dominant taxa proliferate and are subsequently redeposited into the soil via feces—a process that can appreciably augment microbial diversity (Sun et al., 2021; Wang et al., 2022; Huang et al., 2013; Yang et al., 2024). In addition, earthworms can also spread spores and fungal fragments through their body surface or stomach, thereby affecting the microbial community structure. This is crucial for fungi that form fruiting bodies in the litter layer, as it greatly promotes the release and dissemination of spores (Lilleskov and Bruns, 2005). These soil animals regulate the ratio of bacteria to fungi through their own activities, enhance the functional diversity of microorganisms in soil ecosystems through direct feeding and digestion processes, and also affect the structure of microbial communities, ultimately having a profound impact on soil ecological functions.

It is noteworthy that this reduction in local (α) diversity occurred concurrently with an increase in community stability and complexity at the network level, as discussed below, highlighting that diversity alone is an incomplete metric for assessing ecosystem function.

### 4.5 Reconstruction of microbial co-occurrence networks and its implications

As our results demonstrate, they provide strong support for our second hypothesis. The most profound impact of soil fauna was observed in the architecture of the bacteria-fungi co-occurrence network, which serves as an indicator of community stability and functional potential. The combined earthworm and collembolan treatment (EC) significantly increased the number of nodes and links in the network, indicating higher complexity (Heilbronner et al., 2021; Sun et al., 2021). Such enhanced complexity is widely interpreted as a proxy for greater functional redundancy and resilience to disturbance, because a more interconnected and modular network provides alternative pathways for energy flow and material cycling if some taxa are lost (Berry and Widder, 2014; Lupatini et al., 2014; Xiang et al., 2019).

Different faunal groups modulated these inter-kingdom interactions in distinct ways. Earthworms appear to shift the energy channel toward a faster, bacterial-dominated pathway, which can tilt the network interactions from mutualism toward competition (Yu, 2007). In contrast, collembolans, through their selective grazing on fungi and subsequent dispersal of spores via their cuticle and feces, help maintain a more balanced bacteria:fungi ratio and foster fungal-centric connectivity (Lilleskov and Bruns, 2005). The identification of key taxa, such as the concurrent enrichment of the phosphorus-solubilizing fungus Mortierella in the EC treatment, illustrates how combinatorial faunal interventions can simultaneously optimize both the topological structure and the functional capacity of the soil microbiome (Liao et al., 2013; Xia et al., 2022). In conclusion, the manipulation of soil fauna offers a promising avenue for targeted management of keystone microbial guilds and for enhancing the stability and functionality of soil ecosystems in degraded lands.

## 5 Conclusion

Our study provides the first experimental evidence that soil-faunal inoculation acts as a targeted ecological disturbance capable of steering the soil microbiome, thereby offering a concrete biological option for rehabilitating coal-mine wastelands. Two main findings emerge:

1. Distinct faunal treatments significantly rewired microbial community structure and composition.
2. The combined treatment of earthworms and collembolans (EC) representing higher soil faunal diversity outperformed single-fauna additions (C or E) in enhancing microbial community stability. This finding indicates that increased soil faunal diversity can effectively stabilize microbial community structure, which carries practical implications for applied scenarios. Additionally, this outcome opens promising future research avenues, such as exploring optimal soil faunal diversity configurations to maximize microbial community stability across different soil ecosystems.

Network analyzes further revealed a positive relationship between the abundance of soil animals and the number of keystone microbial taxa, indicating that manipulating faunal density provides a lever with which to regulate critical microbial hubs and accelerate the recovery of soil functions. Consequently, introducing soil fauna to derelict mine soils can rapidly redirect microbial assemblages and shorten the ecological-restoration trajectory.

## Data Availability

The data presented in this study are publicly available. The fungal ITS2 sequencing data are available under BioProject PRJNA1404437 in the NCBI Sequence Read Archive (SRA), with representative run accessions SRR36920837, SRR36920834, and SRR36920832. The bacterial 16S rRNA sequencing data are available under BioProject PRJNA1404245 in the NCBI SRA, with representative run accessions SRR36919778, SRR36919777, and SRR36919774.

## Footnotes

1
www.burkardscientific.co.uk

2
http://denglab.org.cn
