# Correction: Enhancement of soil microbial community stability by earthworms and collembolans in soil from abandoned coal mine land

**DOI:** 10.3389/fmicb.2026.1803716

**Published:** 2026-03-04

**Authors:** Junli Jia, Linghui Chen, Qian Liu, Kai Wang, Kang Zhao, Xiaoyu Ren, Xue Gao, Jianmei An

**Affiliations:** School of Life Science, Shanxi Province Engineering Research Center of Protection and Utilization of Endemic Animal Resources, Shanxi Normal University, Taiyuan, Shanxi, China

**Keywords:** edaphic macro-microfauna, microbial community co-occurrence network, mine soil rehabilitation, soil fauna, soil microbiome

Author Jianmei An was erroneously spelled as Jianmei Ani.

The order of authors in the author list of the published paper was erroneously given as:

Junli Jia^†^, Linghui Chen^†^, Qian Liu^*†^, Kai Wang, Kang Zhao, Xiaoyu Ren, Xue Gao and Jianmei Ani^*^

The correct author list reads:

Junli Jia^†^, Linghui Chen^†^, Qian Liu^*^, Kai Wang, Kang Zhao, Xiaoyu Ren, Xue Gao and Jianmei An^*^

Author Qian Liu was erroneously assigned as equal contributing author.

The copyright for this article was incorrectly written as “Copyright © 2026 Jia, Chen, Liu, Wang, Zhao, Ren, Gao and Ani.”

The correct statement is “Copyright © 2026 Jia, Chen, Liu, Wang, Zhao, Ren, Gao and An.”

The original version of this article has been updated.

