# Supplementary material for: Enhancement of soil microbial community stability by earthworms and collembolans in soil from abandoned coal mine lands
Source: Front Microbiol. 2026 Feb 3;17:1636784. doi: 10.3389/fmicb.2026.1636784 (PMC12910935; doi:10.3389/fmicb.2026.1636784)
Supplement: Supplementary file 1 [file Supplementary_file_1.docx]

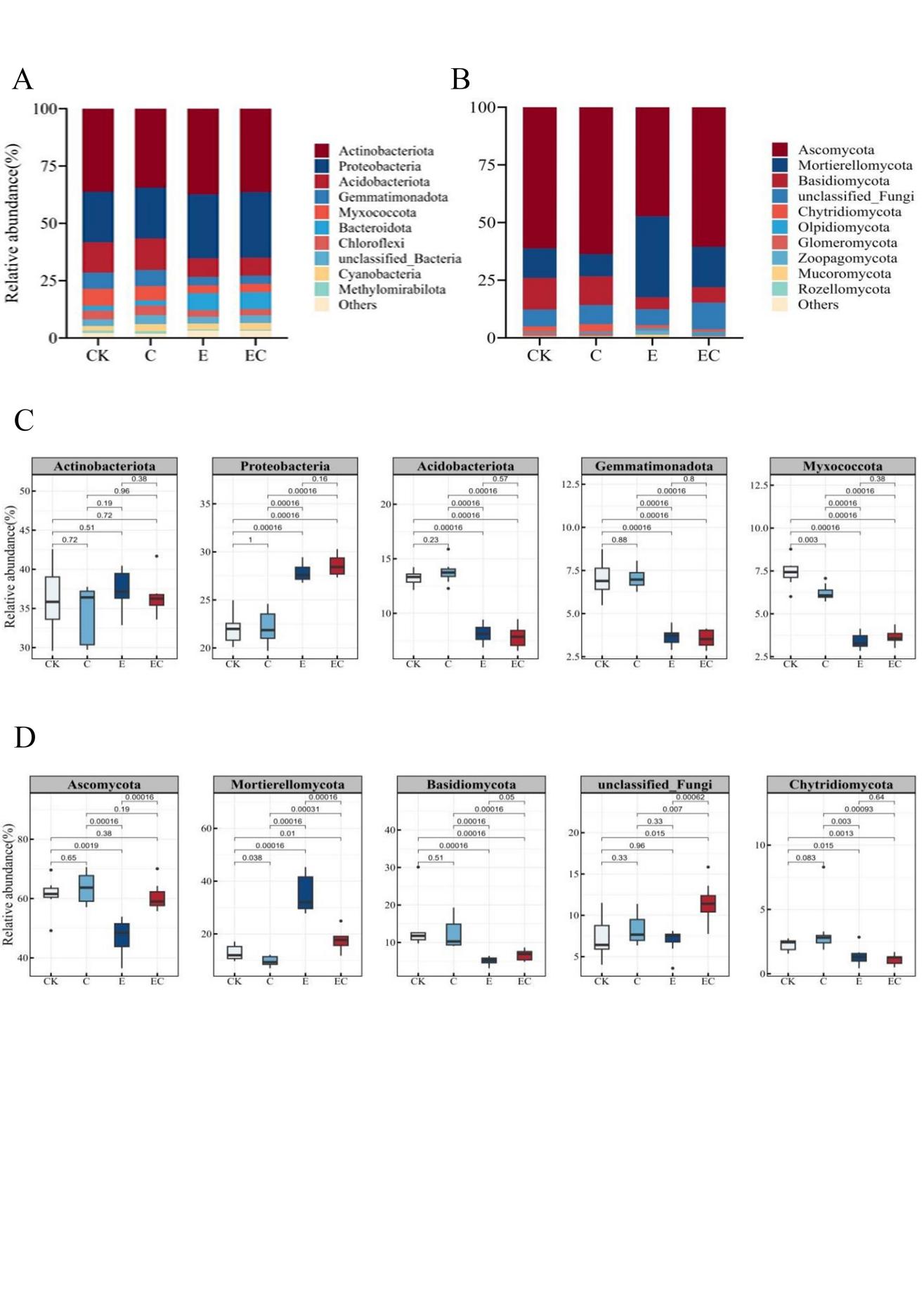


**Figure S1 The community composition of bacteria at the phylum level and the difference between groups of bacteria (A and C) and the community composition of fungi at the phylum level and the difference between groups of fungi (B and D) were tested under different treatments.** The number on the horizontal line represents the P-value.


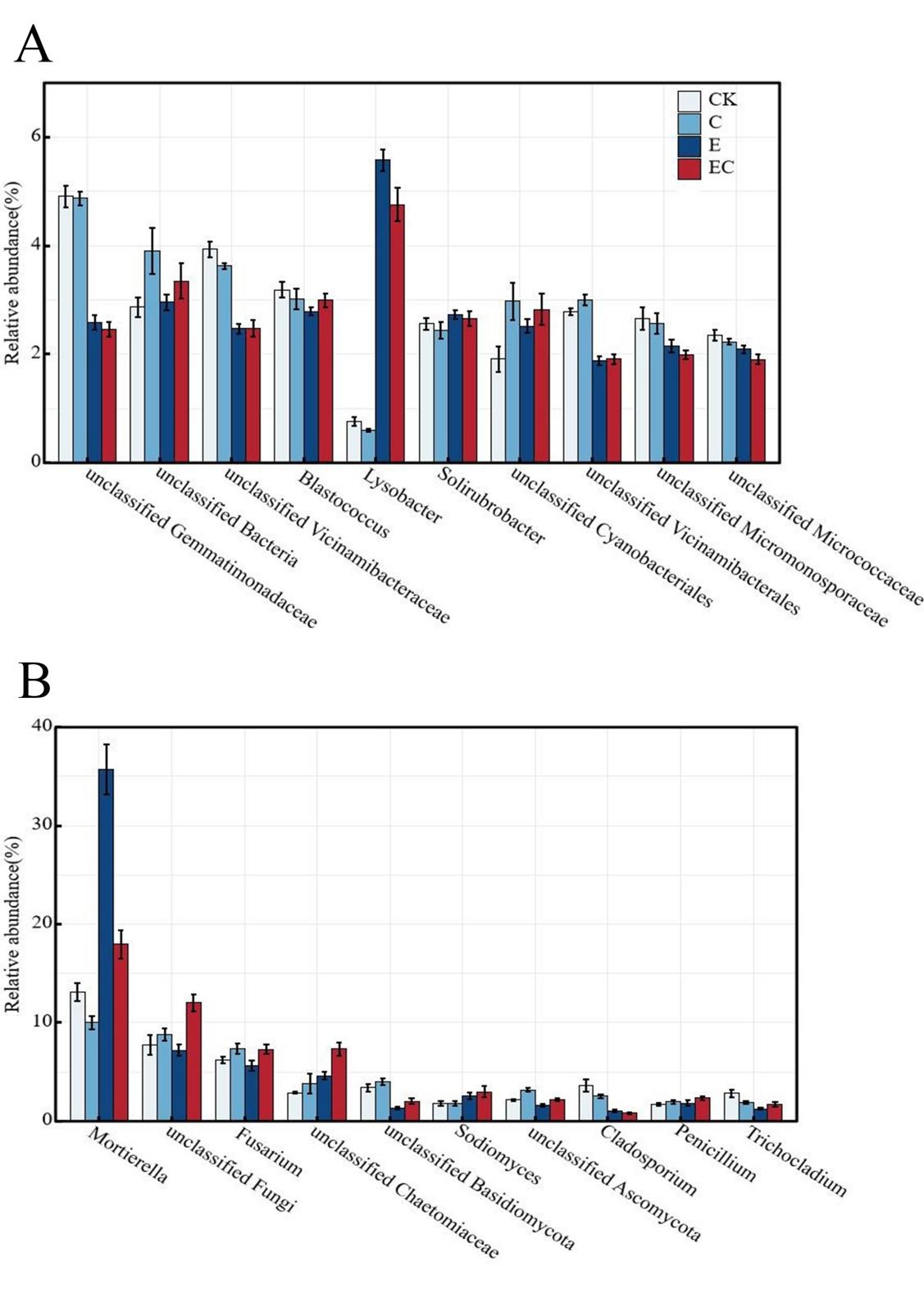


Figure S2 **The composition and relative abundance of bacteria (A) and fungi (B) at the genus level changed between treatments.**

**
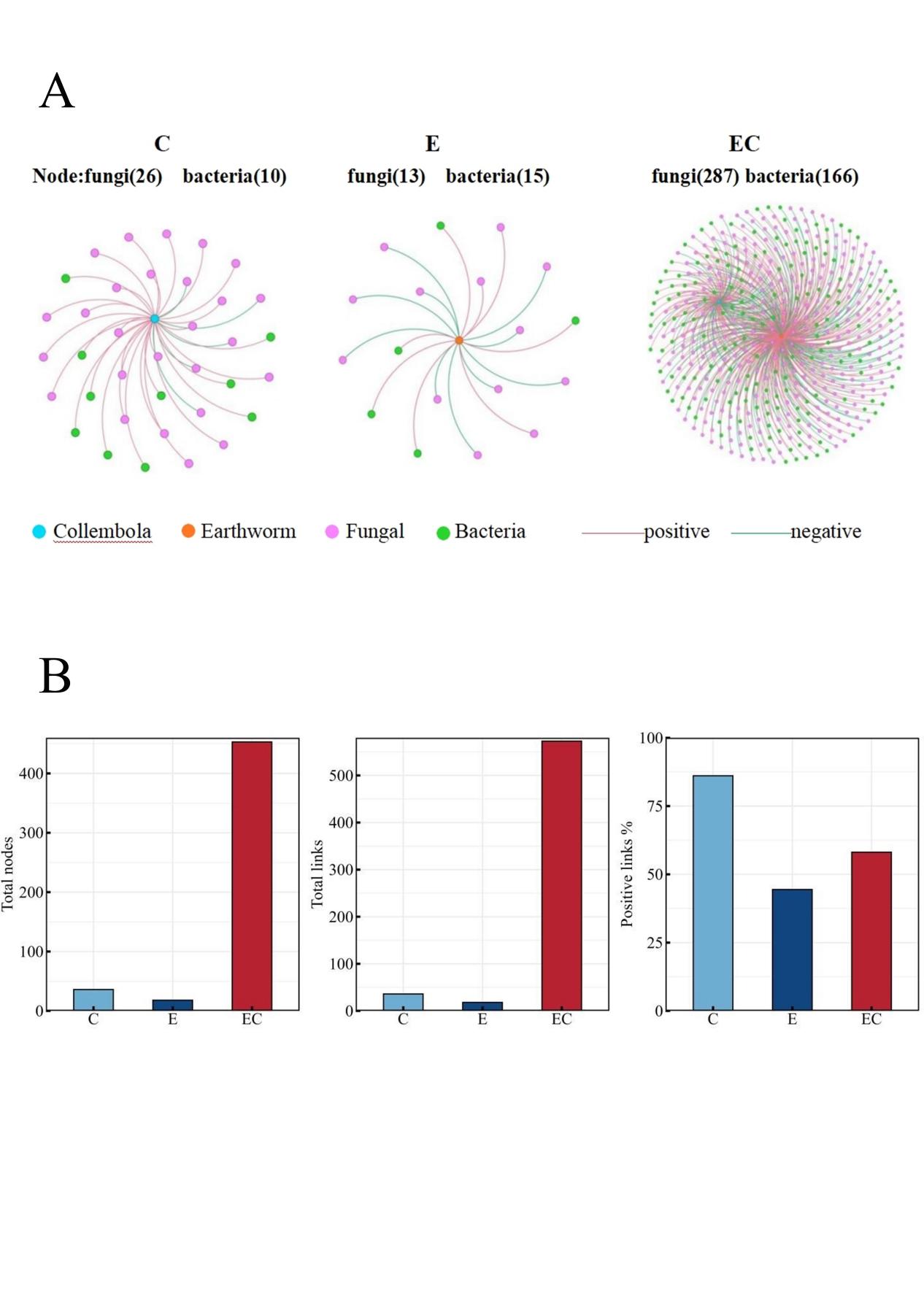
**

Figure S3 **Sub-networks related to soil animals under different treatments and their network characteristics. A:** Build sub-networks using node and edge properties associated with soil animals, with nodes for bacteria, fungi, and soil animals represented in different colors. **B:** Network attributes calculated from soil animal correlation sub-networks.

Table S1 ANOVA analysis of bacterial abundance significance

|  | sum of squares | degree of freedom | mean square | F | p value |
| --- | --- | --- | --- | --- | --- |
| Between-group | 0.002 | 3 | 0.001 | 0.965 | 0.423 |
| Within-group | 0.017 | 28 | 0.001 |  |  |
| Total | 0.019 | 31 |  |  |  |

*represent P<0.05, ** represent P<0.01, *** represent P<0.001.

Table S2 ANOVA analysis of fungal abundance significance

|  | sum of squares | degree of freedom | mean square | F | p value |
| --- | --- | --- | --- | --- | --- |
| Between-group | 0.013 | 3 | 0.004 | 6.302 | 0.002* |
| Within-group | 0.020 | 28 | 0.001 |  |  |
| Total | 0.033 | 31 |  |  |  |

*represent P<0.05, ** represent P<0.01, *** represent P<0.001.

Table S3 PERMANOVA analysis of bacteria communities at different treatments

| Treatment | C | E | EC |
| --- | --- | --- | --- |
| CK | 0.0857** | 0.2947** | 0.2970** |
| C |  | 0.2831** | 0.2796** |
| E |  |  | 0.0910** |

*represent P<0.05, ** represent P<0.01, *** represent P<0.001.

Table S4 PERMANOVA analysis of fungi communities at different treatments

| Treatment | C | E | EC |
| --- | --- | --- | --- |
| CK | 0.0936** | 0.2803** | 0.2976** |
| C |  | 0.3069** | 0.2969** |
| E |  |  | 0.1627** |

*represent P<0.05, ** represent P<0.01, *** represent P<0.001.

Table S5 Topological parameters of bacteria-fungi Co-occurrence network under different treatments

| Treatment | CK | C | E | EC |
| --- | --- | --- | --- | --- |
| Empirical network | | | | |
| Modularity | 0.78 | 0.76 | 0.86 | 0.25 |
| Connectance | 0.04 | 0.05 | 0.04 | 0.11 |
| Web asymmetry | -0.18 | -0.28 | -0.01 | -0.11 |
| Links per species | 2.35 | 3.18 | 1.78 | 10.44 |
| Cluster coefficient | 0.03 | 0.04 | 0.04 | 0.07 |
| Nestedness | 7.57 | 7.66 | 6.37 | 7.78 |
| Specialisation asymmetry | 0.09 | 0.07 | 0.11 | 0.10 |
| Linkage density | 8.47 | 12.63 | 5.79 | 42.74 |
| Interaction evenness | 0.67 | 0.70 | 0.64 | 0.79 |
| Number of species Bacteria | 93 | 95 | 93 | 178 |
| Number of species Fungi | 133 | 170 | 94 | 220 |
| Total links | 7248 | 6433 | 4990 | 24011 |
| Negative edges percentage (%) | 42.60% | 37.56% | 50.92% | 55.28% |
| Average degree | 4.708 | 6.355 | 3.561 | 20.879 |
| Cluster coefficient Bacteria | 0.04 | 0.05 | 0.04 | 0.11 |
| Cluster coefficient Fungi | 0.04 | 0.05 | 0.04 | 0.11 |
| Niche overlap Bacteria | 0.05 | 0.06 | 0.05 | 0.14 |
| Niche overlap Fungi | 0.04 | 0.06 | 0.03 | 0.11 |
| Functional complementarity^Bacteria^ | 166.08 | 185.35 | 127.01 | 609.47 |
| Functional complementarityFungi | 175.46 | 203.46 | 132.72 | 696.56 |
| Random Networks | | | | |
| Modularity | 0.44±0.01 | 0.35±0.00 | 0.54±0.01 | 0.15±0.00 |
| Connectance | 0.04±0.00 | 0.05±0.00 | 0.04±0.00 | 0.11±0.00 |
| Netedness | 6.20±0.38 | 6.24±0.34 | 5.97±0.39 | 6.15±0.21 |

Robustness was measured by the proportion of species remaining in the community after percent of nodes were randomly removed. The t test was used to measure differences in robustness of networks. Different lowercase letters represent significant differences (P<0.05).

Table S6 Key nodes in the bacteria-fungi Co-occurrence network under different treatments

| Treatment | Topological role | Kingdom | Phylum | Class | Order | Family | Genus |
| --- | --- | --- | --- | --- | --- | --- | --- |
| CK | Module hubs | Bacteria | Actinobacteriota | Thermoleophilia | Gaiellales | Gaiellaceae | *Gaiella* |
| C | Module hubs | Bacteria | Proteobacteria | Alphaproteobacteria | Sphingomonadales | Sphingomonadaceae | *Altererythrobacter* |
|  | Module hubs | Bacteria | Gemmatimonadota | Gemmatimonadetes | Gemmatimonadales | Gemmatimonadaceae | *Gemmatimonadaceae* |
| E | Module hubs | Bacteria | Proteobacteria | Alphaproteobacteria | Sphingomonadales | Sphingomonadaceae | *Altererythrobacter* |
|  | Module hubs | Fungi | Mortierellomycota | Mortierellomycetes | Mortierellales | Mortierellaceae | *Mortierella* |
|  | Module hubs | Fungi | Mortierellomycetes | Mortierellales | Mortierellaceae | Mortierellaceae | *Mortierella* |
|  | Module hubs | Fungi | Basidiomycota | Agaricomycetes | Agaricales | Entolomataceae | *Entoloma* |
| EC | Connect or hubs | Bacteria | Proteobacteria | Alphaproteobacteria | Rhizobiales | Xanthobacteraceae | *Bradyrhizobium* |
|  | Connect or hubs | Bacteria | Proteobacteria | Alphaproteobacteria | Rhizobiales | Xanthobacteraceae | *Xanthobacteraceae* |
|  | Connect or hubs | Bacteria | Proteobacteria | Alphaproteobacteria | Rhizobiales | Rhizobiaceae | *Allorhizobium*  *Neorhizobium*  *Rhizobium* |
|  | Connect or hubs | Bacteria | Proteobacteria | Alphaproteobacteria | Rhizobiales | Beijerinckiaceae | unclassified |
|  | Connect or hubs | Bacteria | Proteobacteria | Alphaproteobacteria | Rhizobiales | Labraceae | *Labrys* |
|  | Connect or hubs | Bacteria | Proteobacteria | Alphaproteobacteria | Sphingomonadales | Sphingomonadaceae | *Sphingomonas* |
|  | Connect or hubs | Bacteria | Proteobacteria | Alphaproteobacteria | Sphingomonadales | Sphingomonadaceae | *Ellin6055* |
|  | Connect or hubs | Bacteria | Proteobacteria | Alphaproteobacteria | Dongiales | Dongiaceae | *Dongia* |
|  | Connect or hubs | Bacteria | Proteobacteria | Alphaproteobacteria | Tistrellales | Geminicoccaceae | *Candidatus Alysiosphaera* |
|  | Connect or hubs | Bacteria | Proteobacteria | Alphaproteobacteria | Acetobacterales | Acetobacteraceae | unclassified |
|  | Connect or hubs | Bacteria | Proteobacteria | Gammaproteobacteria | Burkholderiales | SCI84 | unclassified |
|  | Connect or hubs | Bacteria | Proteobacteria | Gammaproteobacteria | Burkholderiales | Comamonadaceae | *Variovorax* |
|  | Connect or hubs | Bacteria | Proteobacteria | Gammaproteobacteria | Xanthomonadales | Xanthomonadaceae | *Lysobacter* |
|  | Module hubs | Bacteria | Proteobacteria | Gammaproteobacteria | Xanthomonadales | Xanthomonadaceae | *Arenimonas* |
|  | Connect or hubs | Bacteria | Proteobaceria | Gammaproteobacteria | Pseudomonadales | Endozoicomonadaceae | *Endozoicomonas* |
|  | Connect or hubs | Bacteria | Actinobacteriota | Actinobacteria | Micrococcales | Micrococcaceae | unclassified |
|  | Connect or hubs | Bacteria | Actinobacteriota | Actinobacteria | Micrococcales | Microbacteriaceae | *Agromyces* |
|  | Connect or hubs | Bacteria | Actinobacteriota | Actinobacteria | Micrococcales | Intrasporangiaceae | unclassified |
|  | Connect or hubs | Bacteria | Actinobacteriota | Actinobacteria | Frankiales | Geodermatophilaceae | *Blastococcus* |
|  | Connect or hubs | Bacteria | Actinobacteriota | Actinobacteria | Frankiales | Geodermatophilaceae | *Blastococcus* |
|  | Connect or hubs | Bacteria | Actinobacteriota | Actinobacteria | Micromonosporales | Micromonosporaceae | *Catellatospora* |
|  | Connect or hubs | Bacteria | Actinobacteriota | Actinobacteria | Propionibacteriales | Nocardioidaceae | *Nocardioides* |
|  | Connect or hubs | Bacteria | Actinobacteriota | Actinobacteria | Corynebacteriales | Mycobacteriaceae | *Mycobacterium* |
|  | Connect or hubs | Bacteria | Actinobacteriota | Actinobacteria | Streptomycetales | Streptomycetaceae | *Streptomyces* |
|  | Connect or hubs | Bacteria | Actinobacteriota | Acidimicrobiia | Microtrichales | Ilumatobacteraceae | *Ilumatobacter* |
|  | Connect or hubs | Bacteria | Actinobacteriota | Thermoleophilia | Solirubrobacterales | Solirubrobacteraceae | *Solirubrobacter* |
|  | Connect or hubs | Fungi | Ascomycota | Sordariomycetes | Hypocreales | Clavicipitaceae | *Metarhizium* |
|  | Connect or hubs | Fungi | Ascomycota | Sordariomycetes | Hypocreales | unidentified | *Hapsidospora* |
|  | Connect or hubs | Fungi | Ascomycota | Sordariomycetes | Hypocreales | Nectriaceae | *Fusarium* |
|  | Connect or hubs | Fungi | Ascomycota | Sordariomycetes | Hypocreales | Clavicipitaceae | *Metarhizium* |
|  | Connect or hubs | Fungi | Ascomycota | Sordariomycetes | Sordariales | Chaetomiaceae | *Acrophialophora* |
|  | Module hubs | Fungi | Ascomycota | Sordariomycetes | Sordariales | Chaetomiaceae | unclassified |
|  | Connect or hubs | Fungi | Ascomycota | Sordariomycetes | Sordariales | Chaetomiaceae | *Condenascus* |
|  | Connect or hubs | Fungi | Ascomycota | Eurotiomycetes | Eurotiales | Aspergillaceae | *Aspergillus* |
|  | Module hubs | Fungi | Ascomycota | Eurotiomycetes | Eurotiales | Aspergillaceae | *Aspergillus* |
|  | Connect or hubs | Fungi | Ascomycota | Eurotiomycetes | Eurotiales | Aspergillaceae | *Penicillium* |
|  | Module hubs | Fungi | Ascomycota | Eurotiomycetes | Onygenales | Onygenaceae | *Auxarthron* |
|  | Connect or hubs | Fungi | Ascomycota | Eurotiomycetes | Chaetothyriales | unclassified | unclassified |
|  | Connect or hubs | Fungi | Ascomycota | Dothideomycetes | Pleosporales | Cucurbitariaceae | *Pyrenochaeta* |
|  | Connect or hubs | Fungi | Ascomycota | Dothideomycetes | Pleosporales | Phaeosphaeriaceae | *Paraphoma* |
|  | Connect or hubs | Fungi | Ascomycota | Dothideomycetes | Pleosporales | unclassified | unclassified |
|  | Connect or hubs | Fungi | Ascomycota | Leotiomycetes | Helotiales | Helotiaceae | *Tetracladium* |
|  | Connect or hubs | Fungi | Ascomycota | Saccharomycetes | Saccharomycetales | Dipodascaceae | *Galactomyces* |
|  | Connect or hubs | Fungi | Ascomycota | unidentified | unidentified | unidentified | unidentified |
|  | Connect or hubs | Fungi | Ascomycota | unclassified | unclassified | unclassified | unclassified |
|  | Connect or hubs | Fungi | Ascomycota | unclassified | unclassified | unclassified | unclassified |
|  | Module hubs | Fungi | Basidiomycota | Microbotryomycetes | Kriegeriales | Camptobasidiaceae | *Glaciozyma* |
|  | Connect or hubs | Fungi | Basidiomycota | Geminibasidiomycetes | Geminibasidiales | Geminibasidiaceae | *Basidioascus* |
|  | Connect or hubs | Fungi | Basidiomycota | unclassified | unclassified | unclassified | unclassified |
|  | Connect or hubs | Fungi | Basidiomycota | unclassified | unclassified | unclassified | unclassified |
|  | Connect or hubs | Fungi | Mortierellomycota | Mortierellomycetes | Mortierellales | Mortierellaceae | *Mortierella* |
|  | Connect or hubs | Fungi | Mortierellomycota | Mortierellomycetes | Mortierellales | Mortierellaceae | *Mortierella* |
|  | Connect or hubs | Fungi | Chytridiomycota | GS14 | unidentified | unidentified | unidentified |
|  | Connect or hubs | Fungi | unclassified | unclassified | unclassified | unclassified | unclassified |
|  | Connect or hubs | Fungi | unclassified | unclassified | unclassified | unclassified | unclassified |
|  | Connect or hubs | Fungi | unclassified | unclassified | unclassified | unclassified | unclassified |
|  | Connect or hubs | Fungi | unclassified | unclassified | unclassified | unclassified | unclassified |
|  | Connect or hubs | Fungi | unclassified | unclassified | unclassified | unclassified | unclassified |
|  | Connect or hubs | Fungi | unclassified | unclassified | unclassified | unclassified | unclassified |

The keystone taxa are module hubs and connectors that are identified based on their within-module connectivity (Zi>2.5) and among-module connectivity (Pi>0.62).

Table S7 The key Genus were correlated with soil animal abundance.

| Kingdom | Genus |  |  | EC | |
| --- | --- | --- | --- | --- | --- |
|  |  | C | E | Earthworm | Collembola |
|  | *Bradyrhizobium* | 0.1448 | 0.013 | 0.1433 | 0.0227 |
|  | *Allorhizobium Neorhizobium Pararhizobium Rhizobium* | / | / | 0.001 | 0.156 |
|  | unclassified Beijerinckiaceae | / | 0.2055 | 0.0015 | **0.316** |
|  | unclassified Xanthobacteraceae | / | 0.0099 | 0.0084 | **0.4271** |
|  | *Labrys* | 0.0203 | / | 0.0003 | **0.5294** |
|  | *Sphingomonas* | 0.013 | / | 0.2615 | 0.0491 |
|  | *Altererythrobacter* | 0.1636 | 0.2049 | 0.0077 | 0.0931 |
|  | *Ellin6055* | / | / | 0.0956 | 0.071 |
|  | *Candidatus Alysiosphaera* | / | / | 0.1877 | 0.1693 |
|  | *Dongia* | 0.0098 |  | 0.2248 | **0.3077** |
|  | unclassified Acetobacteraceae | / | / | 0.1161 | 0.2666 |
|  | *Variovorax* | / | / | **0.6909** | 0.0762 |
| Bacteria | unclassified SC I 84 | / | / | 0.0027 | 0.2754 |
|  | *Lysobacter* | / | / | 0.2985 | 0.1126 |
|  | *Arenimonas* | / | **0.4381** | 0.0476 | 0.0763 |
|  | *Endozoicomonas* | / | / | 0.0904 | **0.752** |
|  | *Agromyces* | / | / | / | **0.3416** |
|  | unclassified Intrasporangiaceae | / | 0.136 | 0.0675 | 0.1342 |
|  | *Ilumatobacter* | / | / | 0.0054 | 0.0658 |
|  | unclassified Micrococcaceae | 0.0097 | 0.1718 | 0.2066 | 0.1428 |
|  | *Blastococcus* | 0.0337 | 0.2763 | **0.3269** | 0.0559 |
|  | *Mycobacterium* | / | / | 0.009 | 0.1046 |
|  | *Catellatospora* | / | / | 0.2504 | 0.1797 |
|  | *Streptomyces* | / | / | 0.0608 | 0.0251 |
|  | *Nocardioides* | 0.0295 |  | 0.0046 | 0.1144 |
|  | *Solirubrobacter* | / | / | 0.0528 | 0.0793 |
|  | *Gaiella* | 0.0251 | / | / | 0.1169 |
|  | unclassified Gemmatimonadaceae | 0.0101 | / | 0.0878 | 0.1332 |
|  | *Hapsidospora* | / | / | 0.0045 | 0.0206 |
|  | *Fusarium* | / | / | 0.0165 | 0.2982 |
|  | *Metarhizium* | 0.0135 | / | 0.0779 | 0.2907 |
|  | *Condenascus* | 0.0095 | / | **0.3378** | 0.1207 |
|  | unclassified Chaetomiaceae | / | / | 0.1198 | **0.3934** |
|  | *Acrophialophora* | / | **0.4369** | 0.0139 | **0.4796** |
|  | *Aspergillus* | 0.0138 | 0.2932 | 0.0901 | 0.2418 |
|  | *Penicillium* | / | / | 0.12 | 0.0894 |
|  | unclassified Chaetothyriales | / | 0.0475 | 0.0024 | **0.379** |
|  | *Auxarthron* | / | / | 0.0188 | 0.1597 |
| Fungi | *Pyrenochaeta* | / | / | **0.3252** | 0.0571 |
|  | *Paraphoma* | 0.0119 | / | 0.005 | 0.2854 |
|  | unclassified Pleosporales | 0.0024 | / | 0.04 | 0.1143 |
|  | *Tetracladium* | / | 0.0297 | 0.0005 | / |
|  | *Galactomyces* | 0.0246 | / | 0.0019 | 0.2523 |
|  | unidentified Ascomycota | 0.0011 | / | 0.0012 | 0.1038 |
|  | unclassified Ascomycota | 0.0016 | / | 0.0306 | **0.3647** |
|  | *Entoloma* | 0.0013 | 0.0407 | **0.4083** | 0.0886 |
|  | *Basidioascus* | / | / | 0.0045 | **0.5553** |
|  | *Glaciozyma* | / | / | 0.0141 | **0.5417** |
|  | unclassified Basidiomycota | / | / | 0.0939 | **0.349** |
|  | unidentified GS14 | / | / | 0.0178 | **0.3278** |
|  | *Mortierella* | / | 0.0495 | 0.048 | 0.2409 |
|  | unclassified Fungi | **0.3649** | 0.2688 | 0.0924 | 0.279 |

Bold fonts indicate a correlation coefficient≥0.3.
